# Maximizing Telerehabilitation for Patients With Visual Loss After Stroke: Interview and Focus Group Study With Stroke Survivors, Carers, and Occupational Therapists

**DOI:** 10.2196/19604

**Published:** 2020-10-23

**Authors:** Stephen Dunne, Helen Close, Nicola Richards, Amanda Ellison, Alison R Lane

**Affiliations:** 1 School of Psychology University of Sunderland Sunderland United Kingdom; 2 Population Health Sciences Institute Newcastle University Newcastle upon Tyne United Kingdom; 3 Faculty of Medical Sciences Newcastle University Newcastle upon Tyne United Kingdom; 4 Department of Psychology Durham University Durham United Kingdom

**Keywords:** telerehabilitation, vision, barriers, facilitators, technology

## Abstract

**Background:**

Visual field defects are a common consequence of stroke, and compensatory eye movement strategies have been identified as the most promising rehabilitation option. There has been a move toward compensatory telerehabilitation options, such as the Durham Reading and Exploration (DREX) training app, which significantly improves visual exploration, reading, and self-reported quality of life.

**Objective:**

This study details an iterative process of liaising with stroke survivors, carers, and health care professionals to identify barriers and facilitators to using rehabilitation tools, as well as elements of good practice in telerehabilitation, with a focus on how the DREX package can be maximized.

**Methods:**

Survey data from 75 stroke survivors informed 12 semistructured engagement activities (7 focus groups and 5 interviews) with 32 stroke survivors, 10 carers, and 24 occupational therapists.

**Results:**

Thematic analysis identified key themes within the data. Themes identified problems associated with poststroke health care from both patients’ and occupational therapists’ perspectives that need to be addressed to improve uptake of this rehabilitation tool and telerehabilitation options generally. This included identifying additional materials or assistance that were required to boost the impact of training packages. The acute rehabilitation setting was an identified barrier, and perceptions of technology were considered a barrier by some but a facilitator by others. In addition, 4 key features of telerehabilitation were identified: additional materials, the importance of goal setting, repetition, and feedback.

**Conclusions:**

The data were used to try to overcome some barriers to the DREX training and are further discussed as considerations for telerehabilitation in general moving forward.

## Introduction

Visual impairments occur in about 65% of stroke survivors [1], with a large proportion of these experiencing partial visual field loss [2] (eg, blindness in a part of their visual field). For such individuals, visual restoration is limited [3]. Partial visual loss can be debilitating, with those who experience it having problems completing activities of daily living [4], an unwillingness to leave the home [5], and an increased risk of falling [6]. Individuals also have their driving licenses revoked, further reducing their independence, and can consequently become more isolated, reliant on support, and depressed [5,7-9]. Overall, people with visual field defects demonstrate poorer functional outcome [10], impaired vision-related quality of life [11], and reduced engagement in rehabilitation [12]. With improving stroke survival rates [13], an increasing number of stroke survivors are living with the long-term consequences of partial visual loss. Therefore, providing effective and accessible treatment for such individuals is important in order to significantly reduce their disability.

The uptake of technology among older people is relatively high, with 48% of those aged 55 years or older owning a tablet computer in 2017 and 8.1 million 55- to 64-year-olds in the United Kingdom using apps regularly [14]. Furthermore, telerehabilitation, the provision of rehabilitation services (therapeutic intervention, progress monitoring, education, training, etc) at a distance via electronic information and communication technologies [15], has been identified as a credible, potentially cost-effective future direction for health and social care [16,17]. Despite this, there is still a significant contingent of stroke survivors who distrust telerehabilitation, as they fear reduced quality of care [17]. Previous evidence has shown that motivation is a significant factor in improving engagement with home-based rehabilitation tools [18]. At present, only a limited number of studies exist regarding the effectiveness of telehealth-based digital interventions [19]. For these reasons, it is important to examine factors that could increase their uptake.

The most common form of telerehabilitation employed to address visual loss is computer-based compensatory training, which teaches stroke survivors adaptive eye movement strategies to cope more effectively with visual loss. Durham Reading and Exploration Training (DREX) is one such version of this training, taking the form of a free, multiplatform app and significantly improving visual exploration, reading, and quality of life [20] (see Multimedia Appendix 1 for further details). We report findings from our 2-stage study with stroke survivors, carers, and health professionals, which explored potential barriers and facilitators to the use of DREX and perceptions about key features for telerehabilitation packages. Stage 1 described key findings from a survey identifying the key elements of patients’ stroke experiences. In stage 2, these elements were addressed through a process of liaising with key stakeholders (stroke survivors, carers, and occupational therapists) to understand experiences of this specific tool (DREX) and adjusting the training package in response to the issues identified, thereby improving the potential for training uptake and user experience.

## Methods

### Recruitment

A snowball sample of 75 UK-based stroke survivors with partial visual loss completed the survey (mean age 63.77 years). The survey was distributed in an online format via prominent stroke Facebook groups (n=66; mean age 57.00 years) or as a paper version upon request (n=9; mean age 66.78 years).

Stroke survivors for interviews and focus groups were selected through opportunity sampling based on geographical location (region of North East England). A total of 32 stroke survivors (18 men; aged 43-83 years, mean age 62.28 years), 10 carers (7 women; 41-75 years, mean age 54.70 years), and 24 occupational therapists (19 women; 22-45 years, mean age 31.13 years) were involved in either focus groups (n=7) or interviews (n=5). Engagement activity characteristics are displayed in Table 1.

**Table 1:** Characteristics of stakeholder engagement activity.

Activity	Stroke survivors, n	Carers, n	Occupational therapists, n	Total, n
Focus group A	4	1	N/A^a^	5
Focus group B	3	2	N/A	5
Interview A	1	1	N/A	2
Interview B	1	1	N/A	2
Focus group C	8	N/A	N/A	8
Focus group D	6	2	N/A	8
Focus group E	6	1	N/A	7
Focus group F	N/A	N/A	12	12
Focus group G	N/A	N/A	12	12
Interview C	1	N/A	N/A	1
Interview D	1	1	N/A	2
Interview E	1	1	N/A	2
Total	32	10	24	66

^a^N/A: not applicable.

### Design

This qualitative study was part of a larger study investigating barriers and facilitators in stroke rehabilitation in general. This data subset explores the interaction between DREX and specific issues in relation to poststroke visual field defects. Full project data and materials can be found on the Open Science Framework [21]. This study was divided into 2 stages. In stage 1, a short survey was used to identify the barriers and facilitators from the stroke survivors’ experiences. Stage 1 data informed the second stage, which aimed to quantify the extent of the major themes identified. Interviews and focus groups were conducted with stroke survivors, carers, and occupational therapists in a semistructured manner to understand the specific impact of these barriers and facilitators and the factors perceived as important within telerehabilitation. Stroke survivors were recruited via Stroke Association groups in the northeast of England and could participate in either a focus group or an interview. Participants had to be at least 18 years old and able to give informed consent. Stroke survivors with communication difficulties were not excluded if they had capacity for consent; however, no stroke survivors reported communication difficulties. The information gathered from these activities was used to generate changes to the DREX package to increase accessibility. Ethical approval was provided by the Durham University Psychology Department Ethics Committee and the study was conducted in accordance with the Declaration of Helsinki.

#### Stage 1: Survey

Full survey details can be found in Multimedia Appendix 2. Survey data were analyzed to inform key points of interest for discussion within stage 2.

#### Stage 2: Focus Groups and Interviews

Survey data permitted the construction of a semistructured interview protocol (see Multimedia Appendix 3 for the interview schedule), which probed further information from a number of identified areas. Specifically, stroke survivors and carers responded to questions regarding their everyday difficulties, rehabilitation journey, experiences with technology, motivation, and levels of support. Occupational therapists were also asked about their experiences, but with a greater focus on understanding the barriers and facilitators they experience in supporting stroke survivors and their views on the use of technology in poststroke visual rehabilitation.

Stroke survivor focus groups were carried out at one of 5 venues: Age UK Roundhouse Ashington Stroke Group, Gateshead Stroke Association Stroke Group, Newton Aycliffe Stroke Group, Teesside Stroke Club, and Stockton-On-Tees Happy Talk Stroke Club. Interviews were carried out in a quiet room in the survivor’s home. The 2 focus groups with occupational therapists took place with the stroke teams at the University Hospital of North Tees and Northumbria Hospital. Discussions were audio recorded with consent.

Focus groups were facilitated by the primary author (an experienced qualitative researcher with clinical experience). Initially, the project and focus group aims were outlined and the participants introduced. The notion of facilitators and barriers were explained, and participants were given the opportunity to discuss their own expectations and clarify any miscommunications. Carers were encouraged to let the stroke survivors give their views first but were also given the opportunity to discuss their own experiences. All participants communicated verbally. The focus groups and interviews were semistructured, with 6 open questions used to stimulate discussion and follow-up questions used depending on responses. The 2 focus groups conducted with occupational therapists instead focused on the DREX training package and how it could be improved, current rehabilitation techniques implemented by therapists dealing with poststroke visual loss, and additional materials that could assist therapists. Focus groups and interviews lasted approximately 60 minutes.

Qualitative methods have proved particularly suitable for the analysis and interpretation of focus group data [22]. Thematic analysis was used [23] to work through the data until a small number of themes that described the data set were identified. Data were managed using Microsoft Word. Specific information regarding the data coding process can be found in Multimedia Appendix 4.

## Results

### Stage 1: Survey Data

Stroke survivors identified family, carers, and occupational therapists as best placed to support them in their recovery. Therefore, these stakeholder groups were included as participants in stage 2. Additionally, confidence with technology and the internet was found to decline increasingly with age, while fear of making mistakes rose. Finally, face-to-face support was identified as the most useful factor in rehabilitation. A full discussion of these results can be found in Multimedia Appendix 5.

### Stage 2: Interview Data

#### Qualification and Quantification of Barriers and Facilitators Identified by Participants

There were 2 main themes and several subthemes that were identified by stroke survivors as factors that act as barriers to using telerehabilitation, while 1 main theme with 1 subtheme was identified as a facilitator. In addition, 4 key features were identified by participants for those designing and creating e-therapies for stroke survivors. These themes are presented in Textbox 1 and discussed below, illustrated with verbatim transcripts. Interestingly, technology was seen as both a barrier and a facilitator, so these are discussed together.

**Factors identified by stroke survivors, carers, and occupational therapists as acting as barriers or facilitators in the use of telerehabilitation in stroke and the key features identified as important for telerehabilitation.**

**Barriers**

1. Acute ward rehabilitation
  Limited occupational therapy time
  Acute patient readiness
2. Perceived disadvantages of technology
  Confidence
  Lack of interest
  Lack of personal contact

**Facilitators**

1. Technology and the internet as a perceived advantage
  YouTube

**Key features for rehabilitation**

1. Additional resources
2. Goal setting
3. Feedback
4. Repetition

#### Acute Ward Rehabilitation as a Barrier

Occupational therapists discussed the limited time they have with stroke survivors in acute settings as a barrier in their rehabilitation. Therapists praised the idea of having a form of therapy they could leave with a stroke survivor to use as and when they want without requiring therapist supervision and time:

It’s also good as well because like obviously we can only provide so much time as well with rehab but if we can give the patient that to do when we’re not there then if they’re doing even an hour every other day then its brilliant. Sometimes we give them things to do and think, “Are they going to do it or not?” but something like that they know it’s there, they do it, log on.

However, occupational therapists also identified that even with prior technological experience, the use of technology on acute wards was often too big a jump for many stroke survivors:

I think one of the reasons our use of DREX hasn’t been as much as we’d like or anticipate has partly been the whole problem of how ready they are for some of this in the acute phase, and I think that can run over into the acute phase at home as well. That applies to DREX but also applies to some of the other rehab activities we apply to patients. And it’s about, if possible, increasing the option to grade patients into it. I often find with my patients on the ward, DREX is just too big of a jump for them. That even if they’ve got some tech knowledge beforehand the assessment is a big jump for them straight away.

#### Perceptions of Technology

A key factor discussed across all focus groups was technology, specifically if and how it is used by stroke survivors. There was a clear dichotomy between those who perceived technology as a positive aspect of rehabilitation and those who viewed it negatively.

#### Technology as a Barrier to Telerehabilitation

Within this overarching theme of technology as a barrier, 3 key reasons were identified by stakeholders as to why they felt technology was not useful. The primary subtheme was confidence with technology; a large number of the stroke survivors talked about loss of confidence post stroke, which seriously impacted their life. Those who were previously technophobic reported having little confidence to learn something new:

Facilitator: Would you ever want to use the internet or need to use the internet?

Participant: I don’t think so, no, I don’t really think so. It would be handy for some things but the amount of learning it would take…

In addition to this, even those who were previously comfortable using technology reported becoming less confident after their stroke:

I use it. Far less than I used to though. I used to be really comfortable on the internet but now I just stick to basics of where I’m comfortable and I wouldn’t go exploring online at all now. I just use Amazon, Facebook, that’s it. I haven’t got the wherewithal that I used to. I used to be very switched on. Now I’m only half switched on.

One aspect of confidence that stroke survivors discussed was fear, for example, fear of making a mistake while online. This was also echoed in a carer’s responses, who similarly felt fearful letting their partner online because of the potential ramifications of errors:

Participant: I’m just frightened of making a mistake. I think that’s my biggest worry. One time I would just go on the internet and do my shopping on the internet. Now I’m frightened if I order 19 of something.

Carer: I haven’t got the confidence to let him [use the internet] because if he gets into trouble on it I’m [in trouble]!

A second subtheme was level of interest; some stroke survivors were not interested in accessing technology. This was encapsulated in one participant’s response:

I didn’t have an interest then, I haven’t got an interest now.

It is important to understand that individuals may be reluctant to use technology purely because they have previously not had to use it and do not see a need now.

A third subtheme relating to disadvantages of technology was stroke survivors not appreciating the lack of personal contact. Some stroke survivors viewed the emergence of everything being done online, including rehabilitation, as a barrier in their rehabilitation journey:

The other thing as well is everything is done on the internet, there’s no personal contact. I think sometimes you need to break that barrier down.

#### Technology as a Facilitator for Telerehabilitation

Although some stroke survivors viewed technology as a barrier, others spoke about the benefits of technology and its positive impact:

Technology has been my saviour.

Specific positives were attributed to the accessibility of certain technology. Particular mention was made of YouTube and the advantage of being able to watch an online video repeatedly:

YouTube videos would help teach me stuff because the beauty of YouTube is I can play it again and again and again.

#### Key Features of Telerehabilitation Packages

##### Additional Resources

After discussion with occupational therapists, it became apparent that there was no fixed activity provided for stroke survivors with visual loss to complete on acute wards, resulting in the time-consuming process of therapists creating their own materials. This led to therapists discussing the specific tools and materials they could use:

So that’s something we’re already doing, not in the same format. So it’s more paper based so it doesn’t move up and it’s not…we just make them ourselves as well. It’s not as content heavy, it’s not moving through space the way the app is with having to keep up and that.

One theme that emerged was a need for stroke survivors to be provided with opportunities to better understand the requirements of the training packages used in telerehabilitation and the need for tools that could assist occupational therapists in delivering effective therapy in acute wards.

##### Goal Setting

Stroke survivors described the positive nature of setting goals in their rehabilitation and how this aided their rehabilitation in general:

[I] think to myself I’m going to improve…again it was that 4%, 8%, 10%, 12%.

Generally, goal setting is a good motivator and its inclusion in telerehabilitation provides stroke survivors with further motivation to undertake exercises above and beyond their rehabilitative benefit.

##### Feedback

Feedback was highlighted by occupational therapists as critically important to rehabilitation, with stroke survivors feeling more positive and having a sense of achievement after receiving progress feedback:

[Feedback] gives you that boost, doesn’t it. They can say, “I’m improving.” Because you like to know if you’re improving.

##### Repetition

Stroke survivors discussed the positive aspects of repetition in their rehabilitation. One patient discussed the benefits of being able to do things repeatedly, as with his memory issues, this was the only way he could function independently:

Facilitator: So for you, do you think the repetition…because obviously the tasks themselves are extremely repetitive. You’re doing the same kind of training over and over again. Is that something that works really well for you?

Patient: Yeah because it takes me a while to get a hang of it and then I’m ahead of the game. Repetition is the secret of running my life.

#### Applications of These Findings to DREX

Participant responses highlighted a range of issues in the application of telerehabilitation for stroke survivors with visual field defects. We outline alterations made to the DREX training package in order to directly address these issues.

In liaison with occupational therapists, we created a dossier of paper-based tasks for use by stroke survivors who are not currently able to access the training. The tasks are paper-based versions adapted from the training exercises in the DREX app and provide an intermediary form of compensatory training for acute ward use. This tackles several identified issues, including alleviating some of the time pressure on therapists, since they do not have to do this task anymore, and addressing patient readiness for telerehabilitation by providing a simplified form. It could also be a starting point for those lacking confidence or interest in technology by familiarizing them with the tasks in a different format first. Therapists have begun trialing these resources at several local National Health Service trusts and have found them helpful:

I can imagine a lot of people that it will be a real lifeline for because, you know, we run to the photocopy [sic], get our bits of paper, we make stuff up ourselves for them to do.

Furthermore, working with stroke survivors and therapists, we updated user guides and website information to provide a more accessible service to key stakeholders. These user guides have also been printed and provided to therapists in acute settings to avoid having to use technology to access them, which may be a barrier to some. Furthermore, in accordance with an identified facilitator, a YouTube channel was created that included task demonstration videos. Such additional resources were designed to help alleviate some concerns that users experience regarding unknown technology, and we know that the videos viewed more often are those that correspond to some of the more difficult aspects of navigating the training, indicating that they are a tool that can aid this.

Given the importance of feedback in rehabilitation, the delivery of this within the DREX training was improved; users now receive a speed and accuracy score after every training block. This allows them to effectively track their progress over the course of training. However, we are continuing to investigate ways to improve the feedback provided. Although stroke survivors have responded well to receiving feedback, therapists believed the feedback delivery could be improved. Therapists felt one-to-one monitoring of stroke survivors’ feedback would further help stroke survivors understand how they are progressing and challenge them to continually improve.

I thought it might be quite helpful to sit down with someone and go through it afterwards to say, “How did you find this,” and “Well this is what it’s shown.” “This is what you’ve done really well” or “This part shows that’s what you’re saying is really difficult.” For some people it might be really helpful to have those conversations in some more detail.

Delivery of feedback is an extremely important factor in the success of rehabilitation in clinical populations, and this factor was no less understated in the responses of occupational therapists. This provides an interesting point in the move toward telerehabilitation, as this personalized delivery may be lost.

## Discussion

### Principal Findings

Through interviews with key stakeholder groups involved in stroke survivor rehabilitation, this study identified several facilitators and barriers to telerehabilitation. A general lack of confidence with technology, the perceived fear of using telerehabilitation, and the reduced face-to-face contact that a move toward more technological solutions in rehabilitation would bring were highlighted as the main issues stroke survivors faced. Initial in-person visits are one way to alleviate initial fear, as they can facilitate stroke survivor engagement and motivation in the rehabilitation process [24].

Telerehabilitation has been shown to have positive outcomes in motor consequences of stroke, health outcomes, carer support, and health professional satisfaction, albeit evidence quality was recognized to be poor [25]. The present study, therefore, thoroughly addresses the issues that stroke survivors with visual impairment face when using telerehabilitation.

On the basis of the work presented here, we recommend that several core issues be considered in the development and delivery of telerehabilitation. Compensatory training for poststroke visual field rehabilitation is repetitive in nature, which is why it works. It is important to ensure that such tools provide repetition in the most accessible way to ensure that repetition does not result in disengagement. Feedback and goal setting are important considerations when creating telerehabilitation, as they can improve engagement and motivation, but it is also important to provide a tangible progress update. In DREX, goals are set by the individuals based on feedback received when training. Previous studies have highlighted the motivational benefit of individual goal commitment and goal setting on successful performance in other tasks [26]. As such, introducing goal-setting elements and feedback mechanisms into future telerehabilitation for stroke survivors would be beneficial, especially tailored programs that allow final goals (eg, overall aim) and interim goals (eg, daily target) to be determined.

In order to make telerehabilitation as accessible as possible for the majority of individuals, it would be valuable to consider intermediary steps and additional resources that can aid in intervention delivery. Using a multimodal approach (eg, paper, text, audio, visual elements, etc) enables these resources to be understood and used by all stakeholder populations. Engaging with key users prior to and during the development of telerehabilitation packages can ensure the creation of tools that are of maximum effectiveness and accessibility. For example, if during the acute phase of rehabilitation, perceptions of technology are identified as a barrier, then intermediary steps can be put in place to alleviate concerns and assist in the eventual uptake of the telerehabilitation tool. Additionally, training and education of users and key stakeholders involved in the application of telerehabilitation is an important consideration previously found to lead to more effective treatment [27].

### Conclusions

In summary, this study identified several facilitators and barriers to telerehabilitation from the perspectives of the key stakeholders involved in stroke survivor rehabilitation, and it gathered opinions regarding key features required in telerehabilitation. This process has not only given a voice to the key populations directly involved in the rehabilitation process but also developed an understanding of the most important factors in developing a successful and engaging telerehabilitation tool to improve accessibility and usability. More generally, the issues highlighted by all of the populations engaged with show the need to address the disparity between acute care and the mountainous step to what comes next. An intermediary support phase may negate many of the identified barriers in stroke populations and deflect the fear that telerehabilitation reduces quality of care. Using the mitigations outlined will result in a more personalized recovery program for all stroke survivors.

## Appendix

Multimedia Appendix 1 What is DREX: Compensatory eye-movement training following stroke.

Multimedia Appendix 2 Full survey.

Multimedia Appendix 3 Focus group and interview protocol.

Multimedia Appendix 4 The process of thematic analysis.

Multimedia Appendix 5 Supplementary survey data.

## Abbreviations

DREX: Durham Reading and Exploration
