# Supplementary material for: Maximizing Telerehabilitation for Patients With Visual Loss After Stroke: Interview and Focus Group Study With Stroke Survivors, Carers, and Occupational Therapists
Source: J Med Internet Res. 2020 Oct 23;22(10):e19604. doi: 10.2196/19604 (PMC7647809; doi:10.2196/19604)
Supplement: Multimedia Appendix 1 [file jmir_v22i10e19604_app1.pdf]

Durham Reading and Exploration Training (DREX)

The Problem

One common disabling consequence of brain injury is partial blindness known as a homonymous visual field defect. Most cases result from stroke. People's vision rarely gets better and therefore the majority of individuals experience chronic visual loss which severely impairs daily living. Such patients experience difficulties in everyday tasks such as crossing the road, navigating unfamiliar environments, and shopping independently, and often experience a reduced quality of life which indeed extends to their family. Patients also have their driving licence revoked further reducing their capacity for independent living. As a consequence of such difficulties individuals with this visual loss can become isolated and depressed. However, there is currently no standardised, systematic treatment offered to such patients via the NHS. This is what we have set out to achieve.

What is DREX

Over the past 10 years, we have developed a treatment for visual field defects. Called DREX (Durham Reading and EXploration training), it compensates for visual loss by training patients to move their eyes so that they can cope more effectively with not being able to see a part of their visual field. We have found these simple computer-based visual search tasks, which target both search-based eye movements and reading, improve exploration and vision-related behaviour with significant quality of life benefits. DREX incorporates tasks that combine both reading and exploration. In the exploration training patients are presented with a visual array and have to scan the array to find the odd one out. For example on a single trial patients may see a number of red letter X's randomly placed around the screen. Among this array a letter of a different size, shape or colour will also be present. Users are required to scan the visual array to locate the letter and tap the location at which the odd target out appeared. As the patient improves in their ability to successfully scan the visual arrays and locate the target, the trials become more difficult and challenging with the target being presented further from the centre of the screen and into the user's blind visual field. Furthermore, as the user gets faster at successfully finding the target, the visual array will be presented for a shorter period of time. In this way the app is self-adjusting; increasing with difficulty as the patient learns. In the reading training patients are presented with a word in the centre of the screen. In this type of training patients have to scan the entirety of the word and make a decision about whether or not the word that is presented is a real word or a made-up word. As before, this type of training is self-adapting with the words increasing in difficulty and strings of words being presented to the patient. This type of training not only improves patient's ability to scan the entirety of a word but also improves patients reading accuracy and speed.

Previously we have clinically evidenced the effectiveness of this training in patient populations both in the lab and the patients' own homes (through the provision of a CD from us) [1-2]. More recently we have turned this training into a free, multiplatform app making it as accessible as possible to patients all over the world. We have worked with patients and healthcare professionals to address barriers such as accessibility, technical expertise etc. from the very beginning of its development to ensure its efficacy is not impaired by peripheral, and malleable issues. In this way, patients are able to take control over their own rehabilitation, solving the problem of no current treatment method

for this type of visual loss available on the NHS. We have created a treatment that works, and through the built in assessments, patients can see their improvement thus motivating them to continue. Through our unique clinical portal, healthcare professionals can remotely access patient data, thus personalising care for each patient. Providing an accessible, multi-platform rehabilitation tool where none currently exist on the NHS has been invaluable to a patient population who report feeling mistreated and undervalued.

Associated Links for Further Information

Webpage: https://www.dur.ac.uk/psychology/research/application/drex/

Youtube: https://www.youtube.com/channel/UC4l-rFT4e3KNc3AEPYfBw4A

Twitter: @DREX_Durham

References

1. Aimola L, Lane AR, Smith DT, Kerkhoff G, Ford GA, Schenk T. Efficacy and Feasibility of Home-Based Training for Individuals With Homonymous Visual Field Defects. Neurorehabil Neural Repair 2014 Mar;28(3):207–218. [doi: 10.1177/1545968313503219]
2. Lane AR, Smith DT, Ellison A, Schenk T. Visual exploration training is no better than attention training for treating hemianopia. Brain 2010 Jun 1;133(6):1717–1728. [doi: 10.1093/brain/awq088]
