# Supplementary material for: Maximizing Telerehabilitation for Patients With Visual Loss After Stroke: Interview and Focus Group Study With Stroke Survivors, Carers, and Occupational Therapists
Source: J Med Internet Res. 2020 Oct 23;22(10):e19604. doi: 10.2196/19604 (PMC7647809; doi:10.2196/19604)
Supplement: Multimedia Appendix 2 [file jmir_v22i10e19604_app2.pdf]

Durham University

Thank you for taking part in this survey. The purpose of this research is to build an understanding of the problems that people experience after brain injury, the help with recovery and support that they receive, and also views about technology. This will help us to better understand the barriers that can limit rehabilitation and what we can do to try and improve rehabilitation success.

When filling out this questionnaire please be as honest as possible. All the answers provided in this questionnaire are confidential and you will remain anonymous. If you do not wish to provide an answer or cannot remember the details to any of the questions then please simply move to the next.

Your participation is greatly appreciated. Upon completion of this survey you will be offered the opportunity to be entered into our prize draw to win a £25 Love2shop voucher. To do this, fill out your contact details on the final page. Should you wish to discuss anything about the purpose of this questionnaire or any of the questions then please call Dr Stephen Dunne on **0191 334 0105** or email **s.d.dunne@durham.ac.uk**.

Section 1:- Personal Information

1. Age:

2. Cause of brain injury **(Tick the box that applies to you)**

Stroke: Ischaemic (clot) ☐

Stroke: Haemorrhagic (bleed) ☐

Accident/Injury ☐

Tumour ☐

Other ☐

Don't know ☐

3. Date of brain injury (month/year):

Section 2:- General Information

1. What are the main problems you have experienced since your brain injury occurred? **(Tick all that apply to you)**

Vision ☐

Movement ☐

Balance ☐

Speech ☐

Tiredness ☐

Concentration ☐

Pain ☐

Memory ☐

Emotional changes ☐

Other (Please state)

2. What are the main activities that you think have been affected by your brain injury? **(Tick all that apply to you)**

Reading ☐

Walking ☐

Finding your way around ☐

Shopping ☐

Exercising ☐

Personal Care (Shaving, Washing, etc) ☐

Driving ☐

Crossing the road ☐

Seeing objects ☐

Other (Please state)

3. Do you suffer from any other medical conditions (For example, glaucoma, macular degeneration, arthritis, diabetes, cataracts, etc.)?

Yes ☐

No ☐

If yes, please list these:

4. Who has been involved in your recovery? **(Tick all that apply to you)**

- Occupational Therapist ☐
- Speech and Language Therapist ☐
- Physiotherapist ☐
- Professional Carer ☐
- Nurse ☐
- Doctor (Hospital) ☐
- G.P. ☐
- Family ☐
- Friends ☐
- Social services ☐
- Charity ☐

Other (Please state)

5. Of those you have selected above, who has been the main point of contact for your rehabilitation?

6. **In your opinion**, which of the following people is best placed to help you with your rehabilitation? (Please select only **ONE**)

Occupational Therapist	☐
Speech and Language Therapist	☐
Physiotherapist	☐
Professional Carer	☐
Nurse	☐
Doctor (Hospital)	☐
G.P.	☐
Family	☐
Friends	☐
Social services	☐
Charity	☐

Other (Please state)

7. Do you feel you have had continuous professional help during your rehabilitation?

Yes ☐

No ☐

If yes, from who?

If no, why?

8. Is there support you have not received but that you wanted during your rehabilitation?

Yes ☐

No ☐

Please explain the additional support you would want

Section 3:- Internet Usage

1. How often do you use the internet? **(Tick all that apply to you)**

Everyday ☐

1-3 times a week ☐

Twice a month ☐

Once a month ☐

Never ☐

2. If yes, what do you use the internet for? **(Tick all that apply to you)**

Banking ☐

Emails ☐

News ☐

Using apps ☐

Other (Please state)

☐

3. Do you want to use the internet?

Yes ☐

No ☐

Why?

4. Do you use computers?

Yes ☐

No ☐

Why?

5. Do you use iPads/Android devices?

Yes ☐

No ☐

Why?

6. On a scale of 1-10 (where 1 is strongly disagree and 10 is strongly agree) please rate how much you agree or disagree with the following statements:-

a. I feel confident and comfortable using the internet

Strongly Disagree Strongly Agree

1 2 3 4 5 6 7 8 9 10

b. I feel confident and comfortable using iPad/Android devices

Strongly Disagree Strongly Agree

1 2 3 4 5 6 7 8 9 10

c. I feel confident and comfortable downloading and using apps on Google Play/the Apple Store

Strongly Disagree Strongly Agree

1 2 3 4 5 6 7 8 9 10

d. I am afraid of making a mistake when using the internet

Strongly Disagree Strongly Agree

1 2 3 4 5 6 7 8 9 10

Section 4:- Visual Problems only

Please complete this section if you have experienced any visual problems.

DREX (Durham Reading and Exploration training) is a computer-based training app available for free download. DREX is visual training to help people compensate for the visual problems suffered by many people after brain injury. It is self-adjusting, allowing people to train themselves easily in their own home. It involves a series of tasks that gradually get more difficult promoting the development of more efficient eye-movements and increased visual awareness. Half of the training is also specifically tailored towards improving reading, a common problem associated with visual field loss

1. Have you used the DREX training?

   Yes ☐

   No ☐

2. If you have used DREX training, did you use the computer training or did you use the DREX app?

Computer training ☐

DREX app ☐

Don't know ☐

3. If you have not used DREX training before, would you use the DREX training?

Yes ☐

No ☐

Don't know ☐

4. If no, why would you not use the DREX app? Please state below

5. What could be done to support you using the DREX app?

6. What kinds of support would you find most helpful. Please use the scale below where 1 is not at all helpful and 10 is extremely helpful?

a. Face to face support

Not at all helpful Extremely Helpful

1 2 3 4 5 6 7 8 9 10

b. One to one support over the phone

Not at all helpful Extremely Helpful

1 2 3 4 5 6 7 8 9 10

c. Audio guides

Not at all helpful Extremely Helpful

1 2 3 4 5 6 7 8 9 10

d. User guides online

Not at all helpful Extremely Helpful

1 2 3 4 5 6 7 8 9 10

e. Paper based guide

Not at all helpful Extremely Helpful

1 2 3 4 5 6 7 8 9 10

f. Workshop

Not at all helpful Extremely Helpful

1 2 3 4 5 6 7 8 9 10

g. Online forum

Not at all helpful Extremely Helpful

1 2 3 4 5 6 7 8 9 10

7. Please use the box below to state any type of support we may have missed that you would find helpful

8. Of the support you have selected in the previous question, which **ONE** type of support would you say is most useful for you?

9. Do you have any comments?

Thank you for taking part in this survey. Your views are appreciated. To be entered into our prize draw to win a £25 Love2shop voucher then please leave your details on the following page.

Section 5:- Prize Draw

Thank you for taking the time to complete this survey. To be entered into our prize draw to win a £25 Love2shop voucher then please leave your details below. This is a separate section and your details will not be paired with the answers you have provided to the questions above. Should you win we will use the details provided to contact you

Name	
Email	
Phone	
