# Supplementary material for: Maximizing Telerehabilitation for Patients With Visual Loss After Stroke: Interview and Focus Group Study With Stroke Survivors, Carers, and Occupational Therapists
Source: J Med Internet Res. 2020 Oct 23;22(10):e19604. doi: 10.2196/19604 (PMC7647809; doi:10.2196/19604)
Supplement: Multimedia Appendix 3 [file jmir_v22i10e19604_app3.pdf]

Focus group questions

Introduction

Thank you for participating in this focus group. I am part of a research project called DREX, which is the Durham Reading and Exploration training. The aim of today is to have a discussion about your experiences with stroke and your recovery. What kind of things you find difficult, how you combat these on a daily basis and importantly the things that can be done to assist you. We hope that the information gathered will enable us to improve rehabilitation services in the future. If you all agree we would also like to record this session just in case we miss anything, as that way we can listen back.

It’s important to set up some ground rules just to facilitate the discussion and to make sure that we all get the most out of this group. Firstly it’s important to point out that this discussion will be completely confidential and you will all remain anonymous. Secondly it is important that when one person is talking and giving their views that there is no cross-talking and we remain respectful of each other’s views. Finally, if anyone is spending too much time talking or is stopping others from giving their points of view, I will step in just to keep the discussion moving forward. We appreciate your views and are grateful for your participation in this.

Starter questions

1. Daily problems
    a. What problems do you experience daily
    b. Can you give me examples?

2. What do you find helps you with your rehab? (Facilitators)
    a. Can you give me examples?

3. What do you find hard about your rehab? (Barriers)
    a. Can you give me examples?

4. What is your experience with using/accessing the internet?
    a. How do you feel about the internet/tablets/iPad’s?
    b. What do you like/not like?

5. What problems have you encountered when using the internet/Why don’t you use the internet (Barriers)
    a. Can you give me examples?

6. What kinds of things help when using the internet (Facilitators)
    a. Can you give me examples?

Other themes in case of distraction/discussion off-point

- Support
- Time
- Motivation
