# Supplementary material for: Maximizing Telerehabilitation for Patients With Visual Loss After Stroke: Interview and Focus Group Study With Stroke Survivors, Carers, and Occupational Therapists
Source: J Med Internet Res. 2020 Oct 23;22(10):e19604. doi: 10.2196/19604 (PMC7647809; doi:10.2196/19604)
Supplement: Multimedia Appendix 4 [file jmir_v22i10e19604_app4.pdf]

Process of Thematic Analysis

Thematic analysis was used, working through the data until a small number of themes were identified which described the dataset. Data was managed using Microsoft Word. There were five critical stages: identifying initial codes; coding the data set; building themes from identified codes; understanding the relationship between themes; and interpreting these themes. This analysis was an iterative process with overlap between the stages. Data was transcribed by the principal investigator, and during this process initial ideas were noted [1]. The transcribed data was read several times and the recordings listened to several times to ensure transcription accuracy. Data was then coded and explored for themes; these explained larger sections of the data by combining different codes that were considered the same aspect. All initial codes relevant to the research question were incorporated into a theme. Braun and Clarke [2] also suggest the development of thematic maps, and these were used to visualise relationships between themes. At this point any themes that did not have enough data to support them or were too diverse were discarded. This refinement of the themes took place on two levels; primarily with the coded data ensuring they formed a coherent pattern, and secondly once a coherent pattern was formed the themes were considered in relation to the dataset as a whole. This ensured the themes accurately reflected what was evident in the entire data. Further coding also took place at this stage to ensure no codes had been missed in the earlier stages. Once a clear idea of the various themes and how they fitted together emerged, analysis moved to defining and naming the themes. Considerations were made not only of the story told within individual themes, but how these related to the overall story that was evident within the data. The final stage involved choosing transcript examples to illustrate elements of the themes.

References

1. Riessman CK. Narrative analysis. London: Sage; 1993.
2. Braun, V., & Clarke, V. (2006). Using thematic analysis in psychology. *Qualitative Research in Psychology, 3*(2), 77-101.
