# Supplementary material for: Maximizing Telerehabilitation for Patients With Visual Loss After Stroke: Interview and Focus Group Study With Stroke Survivors, Carers, and Occupational Therapists
Source: J Med Internet Res. 2020 Oct 23;22(10):e19604. doi: 10.2196/19604 (PMC7647809; doi:10.2196/19604)
Supplement: Multimedia Appendix 5 [file jmir_v22i10e19604_app5.pdf]

Survey Supplementary Material

Stroke related problems

Respondents' main non-visual complaint post-stroke was tiredness (16.29%), followed by issues with balance (14.39%), concentration (14.02%), movement (13.64%), memory (13.64%) and emotion (13.64%). Fewer participants reported issues with speech (8.71%) and pain (5.68%).

The main activity that participants reported being affected was driving (19.05%). Respondents also reported difficulty with reading (13.39%), seeing objects (13.10%), crossing the road (10.71%), walking (11.61%), exercising (9.52%), finding their way around (8.33%), and shopping (8.33%).

Predictors of post-stroke technological confidence

Four hierarchical regressions were conducted to investigate any predictors of respondent's confidence online and using technology post-stroke (see Table 3 for summary). When considering confidence using the internet, Age, (t = -4.44, p <.001), Speech, (t = -2.80, p =.008), and Driving (t = -2.34, p = .024) were significant predictors. All were negatively associated with confidence; namely younger respondents who did not have issues with speech or driving were more confident using the internet. The final model was significant ($F_{(3, 46)}$ = 11.39, p <.001), and explained 42.6% of the variance.

Age, (t = -3.31, p =.002) and Concentration, (t = -3.14, p =.003) significantly predicted confidence using an iPad. Both predictors were negatively associated with confidence, such that younger people with concentration issues were more confident using an iPad. The final model was significant ($F_{(2, 46)}$ = 8.87, p =.001), and explained 27.8% of the variance.

Age (t = -3.82, p <.001) and Concentration, (t = -2.43, p =.019) also significantly predicted confidence using the App store and Google Play. Both predictors were negatively associated with confidence downloading apps, such that younger people with concentration issues were more confident in this. The final model was significant ($F_{(2, 47)}$ = 9.2, p <.001), and explained 28.1% of the variance.

Finally, Age, (t = 2.69, p =.01), Tiredness, (t =2.99, p =.005), Pain (t = -2.90, p = .006) and Driving (t = 2.26, p =.029) significantly predicted worrying about making mistakes online. Age, Tiredness and Driving problems were positively associated with being more afraid of making mistakes, whilst Pain was negatively associated. As such, younger people who had no problems with tiredness or driving, but did have problems with pain were less afraid of making mistakes on the internet. The final model was significant ($F_{(5, 43)}$ = 6.004, p <.001), and explained 41.1% of the variance.

Table 3: Results of regression analyses indicating key predictors of post-stroke confidence online

Confidence using the internet	*β*	*Confidence using iPad / tablet devices*	*β*	*Confidence downloading apps*	*β*	*Fear of making mistakes online*	*B*

Variables							
Age	*-.50****	*Age*	*-.41***	*Age*	*-.48****	*Age*	*-.35**
Speech	*-.31***	*Concentration*	*-.40***	*Concentration*	*-.30**	*Driving*	*-.40***
Driving	*-.26*					*Tiredness*	*-.37***
						Pain	*-.28**
Note: R^2 = .426		*Note:* R^2 = .278		*Note:* R^2 = .281		*Note:* R^2 = .411	
* *p* <.05<br>** *p* <.01<br>*** *p* <.001							

Predictors of most useful types of support post-stroke

Seven stepwise regressions were conducted to investigate predictors of the most useful types of support for tele-rehabilitation post-stroke (see summary in Table 4). Movement Issues, (t = 2.867, p =.006) and Speech Issues, (t = 2.027, p = .049) significantly predicted perceived helpfulness of Face-to-Face appointments. Both were positively associated with finding face-to-face appointments helpful, such that a person with these impairments would find such appointments more helpful. The final model was significant ($F_{(2, 42)}$ = 9.037, p =.001), and explained 30.1% of the variance.

Confidence using the internet, (t = 2.85, p =.007) and Movement Issues (t = 2.49, p = .017) were significant positive predictors of the perceived helpfulness of phone support.; people who were more confident using the internet and reported mobility issues found one-to-one phone support most helpful. The final model was significant ($F_{(2, 43)}$ = 6.22, p =.004), and accounted for 22.4% of the variance.

Whether individuals were afraid of making mistakes on the internet (t = -4.22, p <.001) significantly predicted the perceived helpfulness of Audio Guides, with a negative association found; the more a person is afraid of making mistakes the more helpful they find audio guides. The final model was significant ($F_{(1, 43)}$ = 17.76, p <.001), and explained 29.2% of the variance.

Issues with Personal Care (t = 2.82, p =.007), Issues with Memory (t = -2.41, p = .02) and Confidence using the Internet (t = 4.002, p <.001) significantly predicted the perceived helpfulness of Online User Guides. Issues with memory was were negatively associated, and Issues with personal care and Confidence using the Internet were positively associated, meaning that people who did not have issues with memory but had issues with personal care and felt more confident using the internet found Online User Guides more helpful. The final model was significant ($F_{(3, 42)}$ = 11.031, p <.001), and explained 44.1% of the variance.

Issues with balance (t = -2.22, p = .032) significantly the predicted perceived helpfulness of Paper-Based Guides, and the association was negative; people who did not have issues with balance found paper-based guides more helpful. The final model was significant ($F_{(1, 42)}$ = 4.94, p =.032), and explained 10.5% of the variance.

Confidence using the Internet (t = 5.51, p <.001) significantly predicted how helpful online forums were perceived in a positive way; the more confident a person is using the internet the more helpful they find online forums. The final model was significant ($F_{(1, 44)}$ = 30.33, p <.001), and explained 40.8% of the variance.

Table 4: Results of regression analyses indicating key predictors most useful type of support post-stroke

Face-to-Face	*Β*	*Phone support*	*β*	*Audio Guides*	*β*	*Online Guides*	*β*	*Paper-based Guides*	*β*	*Online forums*	*β*
Variables											
Movement	*.39***	*Internet Confidence*	*.39***	*Making Mistakes*	*-.54****	*Personal Care issues*	*.33***	*Balance*	*-.33**	*Internet Confidence*	*.639****
Speech	*.28**	*Movement Issues*	*.34**			*Memory Issues*	*-.28**				
						Internet confidence	*.47****				
Note: R^2 = .301		*Note:* R^2 = .224		*Note:* R^2 = .292		*Note:* R^2 = .441		*Note:* R^2 = .105		*Note:* R^2 = .408	

* *p* <.05
** *p* <.01
*** *p* <.001

Predictors of most supportive partnership pivots post-stroke

No significant predictors were found.
